# Multispectral optical metasurfaces enabled by achromatic phase transition

**DOI:** 10.1038/srep15781

**Published:** 2015-10-27

**Authors:** Zeyu Zhao, Mingbo Pu, Hui Gao, Jinjin Jin, Xiong Li, Xiaoliang Ma, Yanqin Wang, Ping Gao, Xiangang Luo

**Affiliations:** 1 State Key Laboratory of Optical Technologies on Nano-Fabrication and Micro-Engineering, Institute of Optics and Electronics, Chinese Academy of Science, Chengdu 610209, China

## Abstract

The independent control of electromagnetic waves with different oscillating frequencies is critical in the modern electromagnetic techniques, such as wireless communications and multispectral imaging. To obtain complete control of different light waves with optical materials, the chromatic dispersion should be carefully controlled, which is however extremely difficult. In this paper, we propose a method to control the behaviors of different light waves through a metasurface which is able to generate achromatic geometric phase. Using this approach, a doughnut-shaped and a solid light spot were achieved at the same focal plane using two light sources with different wavelengths as used in the stimulation emission depletion (STED) microscope system. In order to reveal the full capacity of such method, tight focusing at multiple wavelengths is also represented, where the focal spots of different wavelengths are located at the same position. The results provided here may open a new door to the design of subminiature optical components and integrated optical system operating at multiple wavelengths.

As two-dimensional (2D) metamaterials, metasurfaces have been demonstrated to be able to achieve full control of the amplitudes, phases and polarization states of electromagnetic waves^1^,^2^. With gradient phase retardation across the metasurface, it was shown that the ancient optical law should be recast into a more general form^3^,^4^. Using the metasurface-assisted law of reflection and refraction (MLRR)^4^, the wavefront can be arbitrarily controlled, thus enabling many functionalities, such as beam steering, focusing and imaging^3^,^4^,^5^,^6^,^7^,^8^,^9^,^10^. Besides, by matching the boundary conditions, propagating waves in free-space could be completely converted to bound states including surface plasmons (SPs)^5^,^11^,^12^ and oscillating currents^13^. Since such metasurfaces are much thinner and consequently easier to fabricate than metamaterials, it is believed that the first generation of practical meta-devices will utilize this scheme^14^.

Although metasurfaces are promising to achieve novel functionalities, the current designs are still suffering from many drawbacks. In particular, current metasurfaces are limited by their strong chromatic dispersion^15^, although the phase shift can be designed as independent of the operating frequency^16^,^17^,^18^. The chromatic dispersion may severely limit the utilization of metasurfaces in broadband and multi-spectral applications. For example, it’s well known that STED microscopy is one of the most practical methods to break the diffraction limit^19^, which indicates the resolution of far-field optical imaging can never exceed half the wavelength of the illuminated light. The basic principle of STED microscopy relies on the spatially selective deactivation of fluorophores by utilizing two different light beams with different wavelengths and shapes. Nevertheless, the optical system used in traditional STED fluorescence scanning microscope is complicated. It is highly desired to generate the two kinds of beams with a single element. Besides the STED microscopy, the focal length of traditional metasurface lens is dependent of the operating wavelength^20^, which may be disadvantageous for multispectral imaging.

In order to overcome the chromatic dispersion, several methods have been proposed in recent years. For instance, the dispersion properties of lossy metasurfaces were utilized to achieve broadband near-perfect absorption^13^,^21^. In a similar way, the dispersion of anisotropic metasurfaces could lead to a broadband polarization manipulation^22^,^23^,^24^, which results in the metasurface-assisted law of polarization conversion^4^. Recently, Gu *et al*. demonstrated a plasmonic metasurface lens which is capable of focusing dual-wavelength SPs to the same focal plane^25^. Furthermore, Capasso *et al*. showed that the dispersion of phase shift could also be designed to achieve good focusing properties with the same focal length at several discrete wavelengths or even in a broadband spectrum^26^,^27^. Besides the achromatic focusing, it should also be noted that the metasurface-enabled multi-focus lens can also find promising applications in high-capacity data-storage^28^.

In this paper, we report the design and experimental demonstration of the multispectral metasurfaces based on the spin-orbit interaction in nano-aperture array perforated in an ultrathin metallic screen. Instead of using the dispersion of the metasurface unit, we encoded the information of multi-frequencies into one single metasurface to achieve frequency-selective manipulation. Due to the broadband property of the spin-orbit interaction, we could design metasurface to achieve arbitrary-shaped focal spots at discrete wavelengths. To demonstrate the capacity of this method, we also designed a metasurface lens with the same focal length at discrete wavelengths in the visible spectrum.

## Results

### Theoretical model

**Figure 1:**
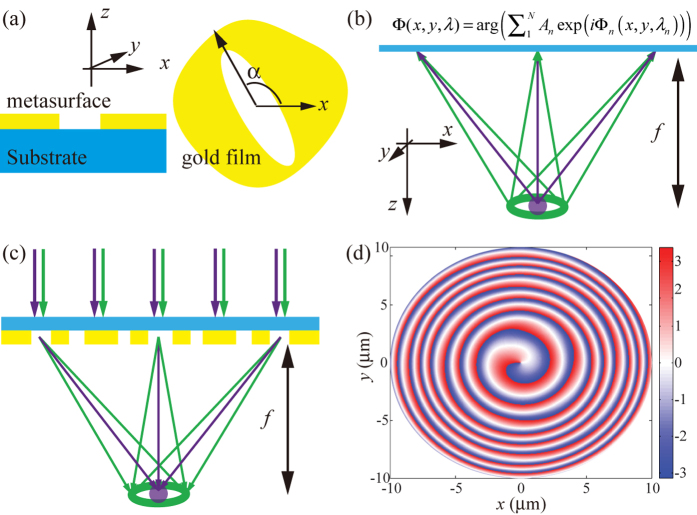
Schematic of the multispectral metasurface based on spin-orbit interaction. (**a**) Unit cell of the nano-aperture. The left and right panels represent the side and top views. The angle between the main axis and the *x*-axis is *α*. (**b**) Schematic of the calculating process of the required phase distribution. (**c**) Generation of a solid and a hollow spot when the designed metasurface is illuminated by light beams with predefined wavelengths. (**d**) Phase distribution designed for a STED lens with focal length of 10 μm.

Space-variant elliptic nano-apertures are the basic elements in the design of our metasurfaces. As illustrated in Fig. 1a, only the polarization perpendicular to the long side is permitted to transmit for the anisotropic structure. Under circularly polarized light (CPL) illumination, each nano-aperture behaves like a polarization filter. Consequently, the space-variant aperture array would result in space-variant polarization states. As a result of the photonic spin-orbit interaction, i.e., the inherent connection between polarization change and phase shift, space-variant phase retardations could be acquired for the handedness-reversed circular polarization^29^,^30^. More specifically, the cross-polarized component of the transmitted light may acquire a phase shift of *φ* = 2*σα*, where *σ* = ±1 represents the left and right handed circular polarization (LCP and RCP), *α* is the inclination angle of the aperture main axis. One can conclude from the equation that the phase shift is independent of the wavelength of the incident light. Consequently, metasurfaces consisting of such nano-apertures could be used in broadband phase modulation.

To achieve multispectral beam manipulation, we resort to the principle of holography^31^, where the information from all angles can be incorporated in a 2D plane. Similarly, we would like to code the information of many wavelengths in a single metasurface. As depicted in Fig. 1b, the phase shift imparted by the metasurface (under plane wave illumination at normal incidence) can be written as:


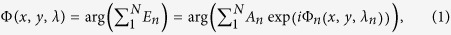


where *E*_*n*_ denotes the complex field, *A*_n_ and Φ_*n*_ are the amplitude and phase of the *n*^th^ virtual source, while *λ*_*n*_ is the corresponding operating wavelength.

### STED lens

What STED needs are a solid focal spot at *λ*_1_ and a hollow spot at *λ*_2_. For *λ*_1_, the complex incident field can be written as:


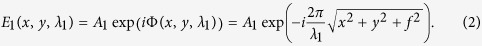


For *λ*_2_, the focused spot incorporates an azimuthal phase gradient in a mathematical form of exp(*ilφ*), where *φ* is the azimuthal angle in the cylindrical coordinate and *l* is the topological charge. As a result, the incident field should be^9^:


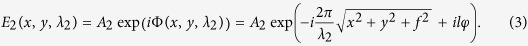


For the simplicity of discussion, we suppose A_1_ = A_2_ = A, thus the required phase distribution for multiwavelength holography is:


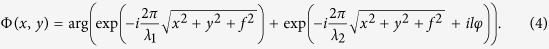


Figure 1c shows that when the designed metasurface is illuminated by normally incident light beams, we would get a solid spot or a hollow spot at the predefined position, depending on the wavelength of light.

**Figure 2:**
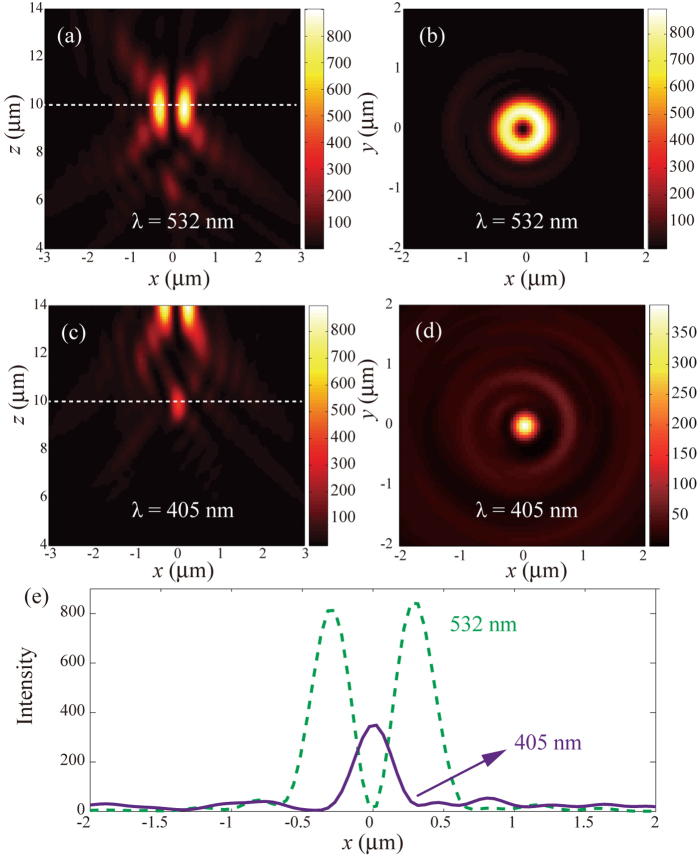
Simulation of the STED lens. (**a**) Numerical results of the vortex beam at λ = 532 nm in the *xz*-plane. (**b**) Intensity in the *xy*-plane at λ = 532 nm and *z* = 10 μm. (**c**) Simulation result for λ = 405 nm in the *xz*-plane. (**d**) Intensity in the *xy*-plane at λ = 405 nm and *z* = 10 μm. (**e**) Intensity distribution along the white lines (*y* = 0) as shown in (**a**,**c**).

In the following, we designed a STED lens using the above principle. The parameters are chosen as λ_1_ = 405 nm, λ_2_ = 532 nm, and *l* = 1. The metasurface lens has a radius of 10 μm, while the observation plane for the solid and hollow spots is set as *z* = 10 μm, i.e. the focal length is *f* = 10 μm. Firstly, the performance of the STED lens was simulated using vectorial diffraction theory^32^. In detail, Fig. 2a,b show the vortex beam (λ_2_ = 532 nm) in the *xz*- and *xy*-plane (*z* = 10 μm), where a clear doughnut-shaped intensity distribution is obtained. Figure 2c,d depict the result at *λ*_1_ = 405 nm in the *xz*- and xy-plane, revealing a solid focus spot at *z* = 10 μm. In order to compare the hollow and solid spots, we plotted the intensity curves along the *x*-direction at the focal plane (Fig. 2e).

Interestingly, we noted that there is also a hollow spot for λ = 405 nm at *z* = 14 μm. This is because the designed metasurface combines the solid spot and the hollow spot functionalities at the same time. Nevertheless, since the hollow spot is shifted along the *z*-direction, it has negligible influence on the final performance.

**Figure 3:**
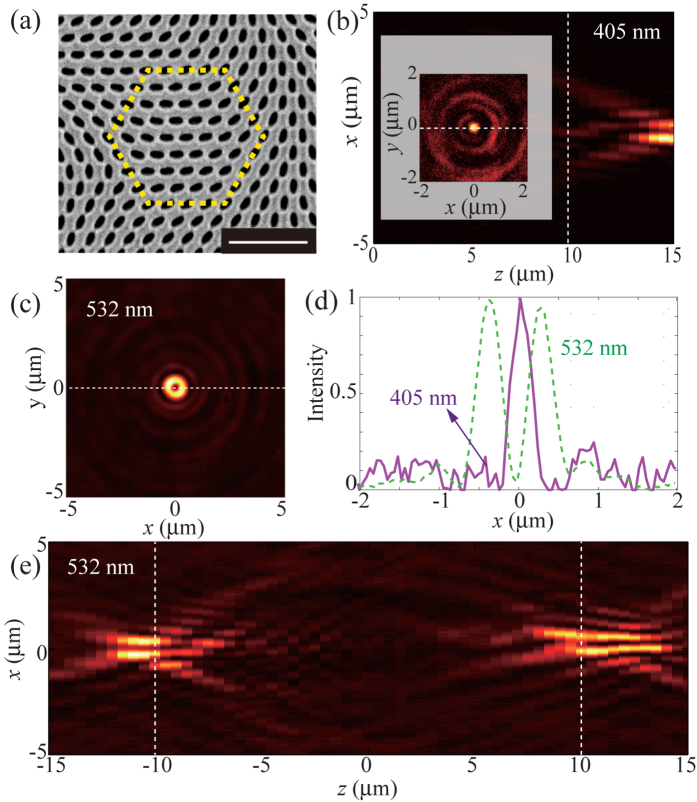
Measurement of the STED lens. (**a**) SEM image of the sample, scale bar: 1 μm. (**b**) Measured intensity distribution in the *xz*-plane at *λ* = 405 nm. Inset shows the solid spot at *z* = 10 μm. (**c**) Measured cross-section of the vortex beam at *z* = 10 μm and λ = 532 nm. (**d**) Comparison of the intensity curves at z = 10 μm and *y* = 0 for λ = 532 and 405 nm. (**e**) Experimental intensity distribution in the *xz*-plane at λ = 532 nm for linear polarization incidence. There are symmetrical hollow spots on both sides at *z* = ±10 μm.

To prove our method and design, a metasurface was fabricated by focus ion beam (FIB) milling a 120 nm thick gold film deposited on a SiO_2_ substrate. The sample has a circular region with a radius of 10 μm. As shown in Fig. 3a, the sizes of the long and short axes of the elliptic nano-aperture are 180 nm and 90 nm, respectively. These apertures are arranged in a hexagonal lattice to increase the symmetry^18^,^21^, with an element-to-element distance of 250 nm.

Subsequently, we measured the light fields generated by the metasurface via a home-made microscope. In the experiment, the incident light is converted to circular polarization via the combination of a linear polarizer and a quarter-wave plate. The *z*-axis of the sample is controlled by a motorized stage, and each step of movement is 0.5 μm. The cross-polarized intensity patterns in different *xy*-planes at different *z* were recorded by a charge coupled device (CCD). By picking out the intensity in the transversal line across the beam center on every intensity map, we could get the intensity distribution in the *xz*-plane or *yz*-plane. Figure 3b is the experimental result in the *xz*-plane for LCP incidence at λ = 405 nm. The inset shows that a solid spot is formed at *z* = 10 μm.

Following the same procedure with the measurement at 405 nm but altering the wavelength of incident light beam to 532 nm, we could get a doughnut-shaped intensity pattern at *z* = 10 μm. The experiment result of intensity distribution in the *xz*-plane is shown in Fig. 3c, agreeing well with the expectation of an optical vortex.

As a result of the polarization-dependence of the geometric phase, different circular polarized beams have inverse phase distribution, which can lead to polarization-controlled lensing and imaging^33^,^34^. To demonstrate the polarization-controlled generation of optical vortex, we measured the sample with a linearly polarized light at λ = 532 nm (Fig. 3d). Since a linear polarization could be decomposed into a LCP and a RCP with the same intensity, there would be two hollow spots along the *z*-axis. Besides the hollow spot formed by RCP at *z* = 10 μm, LCP beam would also produce a similar hollow spot at the inverse position, *z* = −10 μm.

As shown in the above results, the metasurface can generate a focused hollow spot and solid spot at the same desired position for two different wavelengths separately. Clearly, the experiment result approves that the designed metasurface is suitable for STED application. However, to realize three dimensional (3D) super-resolution, the hollow light should be designed as an optical bottle beam^19^,^35^.

### Phase holography for multi-wavelength focusing

One of the most promising applications of multi-spectral metasurface is the focusing and imaging at a constant focal distance for different wavelengths. Such a flat lens can also be achieved via a method similar with the STED lens. Supposing that there are several point sources of light with different wavelength, the distribution of phase on the metasurface can be written as:


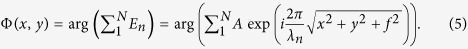


**Figure 4:**
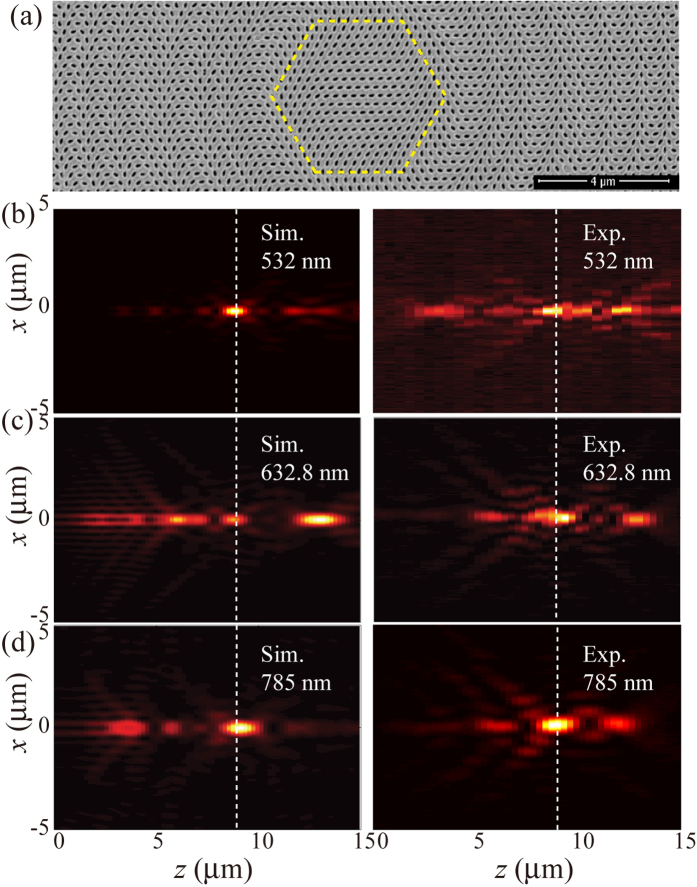
Multispectral metasurface lens. (**a**) SEM image of the fabricated sample. Scale bar: 4 μm. (**b**–**d**) Numerical (left panels) and experimental (right panels) intensity maps for *λ* = 532, 632.8 and 785 nm in the *xz*-plane. The focal length is 9 μm as indicated by the white lines.

**Figure 5:**
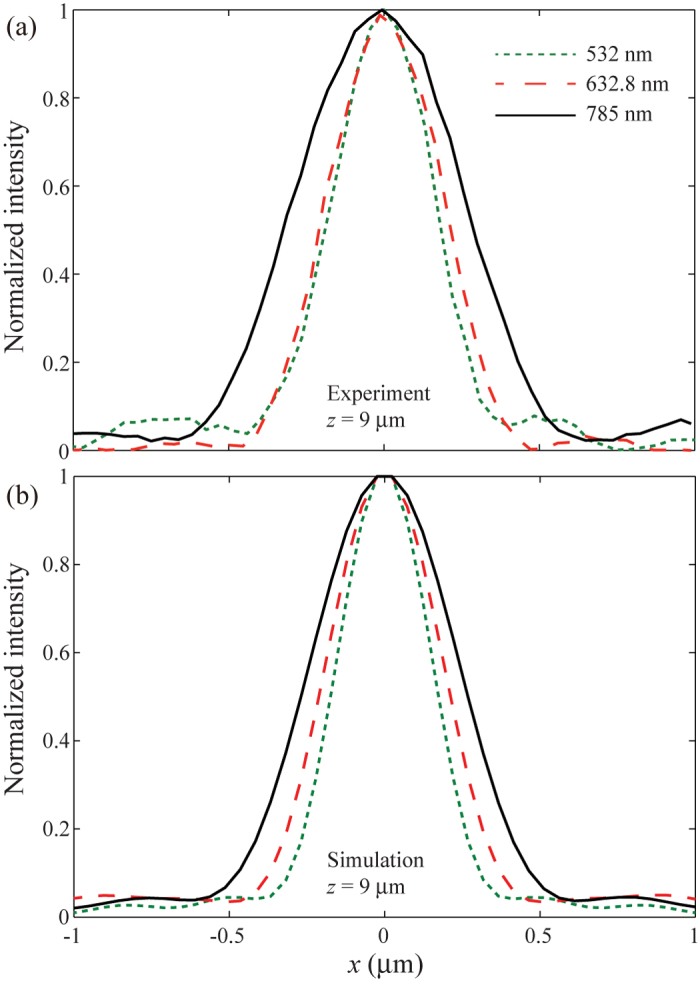
Comparison of the experimental and numerical focusing properties at the focal plane for the multispectral lens. (**a**) Experimental and (**b**) numerical intensity distribution along the *x*-axis for *λ* = 532, 632.8 and 785 nm at *z* = 9 μm.

Once again, we assumed that the amplitudes of these sources are equal. In the design and fabrication of the metasurface, we used three discrete wavelengths, i.e., 532, 632.8 and 785 nm. The radius of the circular sample is 10 μm and the focal length is set as *f* = 9 μm. Figure 4a shows the SEM image of the fabricated sample, where the nano-apertures are arranged similar to the STED lens. We simulated and measured the performance of the metasurface at the three discrete wavelengths. The left panels in Fig. 4b–d display the numerical intensity patterns in the *xz*-plane for *λ* = 532, 632.8 and 785 nm, which qualitatively agree with the experiment results given in the right panels. We compared the full width at half maximum (FWHM) of the numerical and experimental results at *z* = 9 μm for all the three wavelengths. As shown in Fig. 5, this metasurface has the ability of focusing close to the theoretically expected values (or the diffraction limit). By utilizing the metasurface-assisted diffraction theory^4^, it is possible to further obtain achromatic super-resolution imaging at multi-wavelengths.

**Figure 6:**
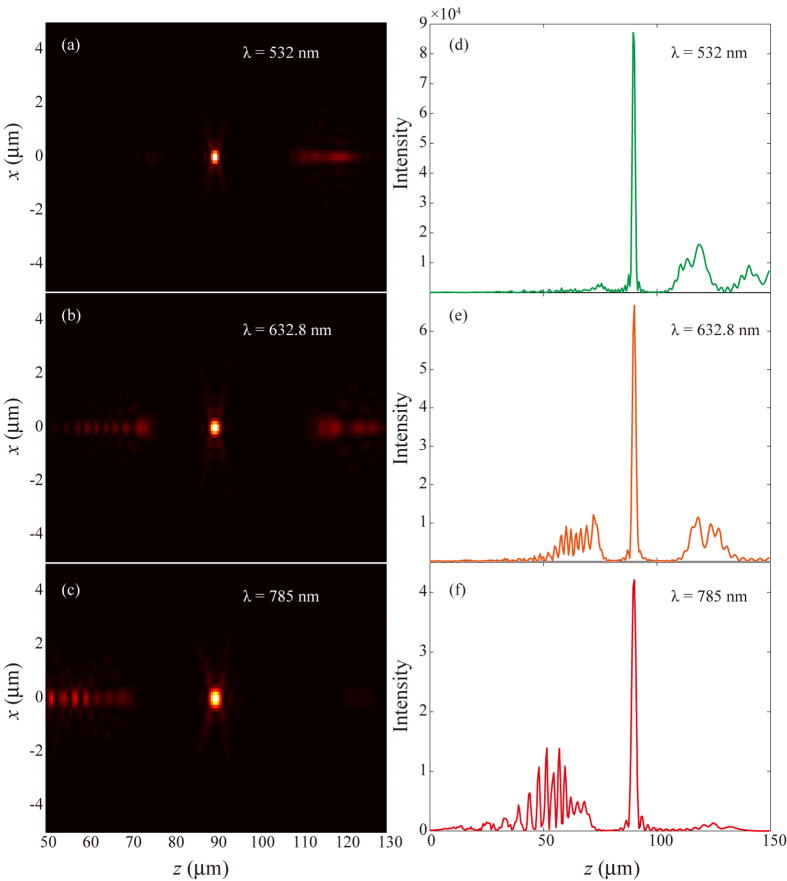
Performance of a multi-focus lens with a relative larger aperture. (**a**–**c**) 2D Intensity in the *xz*-plane for *λ* = 532, 632.8 and 785 nm. (**d**–**e**) Intensity distribution along the *z*-direction at *x* = *y* = 0. The radius of the sample is 100 μm and the focal length is 90 μm, thus the numerical aperture is the same as the sample shown in Fig. 4.

We also noted that the intensity at the focal spot does not coincide with the maximum. A careful study show that this is owing to the finite size of the aperture. In order to reveal this effect, the simulated results for a larger sample with radius of 100 μm and focal length of *f* = 90 μm was given in Fig. 6. Since the numerical aperture of this lens is equal to that in Fig. 4, we can compare the influence of the aperture size. Obviously, as the aperture size increases, the focus performance is improved dramatically, and the intensity maximums always occur at the pre-defined focal length. Furthermore, the light intensity is more than 50000 times larger than the incident wave for all the three wavelengths. Taking λ = 632.8 nm as an example, the maximal intensity for an ideal single-wavelength metasurface lens is 2.2 × 10^5^ times larger than the incident plane wave, thus the corresponding diffraction efficiency exceeds 25%.

In principle, the performance of the multi-wavelength metasurface could be further improved by combining the amplitude and phase modulation techniques. If we divide a metasurface into many regions, and each region is composed of a particular kind of nano-structures that permit only one wavelength to transmit, such metasurface can be used to control different light separately^36^.

## Discussions

In summary, we have proposed an approach to achieve multi-spectral metasurfaces, which can be used to modulate the light fields of different wavelengths. In our approach, we adopted the essence of the holography and encode the phase information of many different wavelengths into one single metasurface. This is different from traditional multi-spectral metamaterials and metasurfaces^26^,^37^, which utilized the complex resonant properties to control the optical responses at different wavelengths.

In order to encode the phase information into surface structures, we utilized the concept of the spin-orbit interaction and geometric phase, which are intrinsically independent of the operational wavelength. To further increase the operating bandwidth, a unique catenary structure could be utilized to eliminate the influence of resonance on the efficiency^32^. We simulated and experimentally demonstrated that the obtained metasurfaces could achieve wavelength-depending focal spots in a realizable way, as well as the multiwavelength achromatic focusing. We believe this concept can be used in the design of subminiature optical components and integrated optical system.

## Methods

### Fabrication

The fabrication of the sample began with depositing a 3 nm thin film of germanium (Ge) on a planar quartz (SiO_2_) substrate which had been cleaned carefully. The germanium was used to improve the adhesion between substrate and Au film. Then a 120 nm film of Au was deposited by magnetron sputtering. Finally, the metasurface consisting of nano-apertures was milled by focus ion beam (FIB, HELIOS Nanolab 650, FEI Company).

### Measurement

All measurements were performed on a home-built microscope. Firstly, the incident collimated beam was converted into CPL via a cascaded polarizer and a quarter-wave plate. The transmitted intensity patterns were then imaged by using a 100× objective lens and a tube lens, and collected by a silicon-based CCD camera (1600 × 1200 pixels, WinCamD-UCD15, DataRay Inc). A quarter-waveplate and a polarizer were used to acquire the cross-polarized components. The incident angle of the laser beam is 0°. The top surface of the gold layer was set at *z* = 0 μm, while the moving step of the motorized stage is 0.5 μm.

## Additional Information

**How to cite this article**: Zhao, Z. *et al*. Multispectral optical metasurfaces enabled by achromatic phase transition. *Sci. Rep*. **5**, 15781; doi: 10.1038/srep15781 (2015).
